# Evaluation of Hematological Indices, Their Longitudinal Trends, and Association With Stratified Dengue Severity in Pediatric Cohorts: A Prospective Observational Study

**DOI:** 10.7759/cureus.115631

**Published:** 2026-09-02

**Authors:** Zoya Tebhla, Ashwini L Chingale, Chinmayi R Joshi

**Affiliations:** 1 Infectious Diseases, Belagavi Institute of Medical Sciences, Belagavi, IND; 2 Community Medicine, Belagavi Institute of Medical Sciences, Belagavi, IND; 3 Pediatrics, Belagavi Institute of Medical Sciences, Belagavi, IND

**Keywords:** dengue thrombocytopenia, hematological parameters in dengue, mean platelet volume, paediatric dengue, platelet indices, prognostic markers in severe dengue, severe dengue management, warning signs dengue

## Abstract

Background: Dengue fever is a widely prevalent viral disease, with one of the most common laboratory findings being thrombocytopenia. Although platelet indices like mean platelet volume (MPV), platelet distribution width (PDW), platelet large cell ratio (P-LCR), and plateletcrit (PCT) are routinely measured, their utility in clinical practice has been limited. This study was planned to evaluate platelet indices across the range of disease severity, understand their importance in terms of their trends over the disease course, and correlate them with other hematological parameters in pediatric dengue.

Methods: A prospective observational study was carried out on a sample of 55 children aged 0-18 years diagnosed with dengue fever in the inpatient pediatrics department. Data regarding clinical features, hematological parameters, including platelet indices, and other laboratory parameters over a period of three days were collected.

Results: A total of 27 (49%) study participants had dengue without warning signs (DF-1), 20 (36.3%) had dengue with warning signs (DF-2), and eight (14.5%) had severe dengue (DF-3). Significantly rising trends of MPV and PDW were observed in the DF-1 group, contrasting with a falling MPV and P-LCR among participants with severe dengue (DF-3). Moreover, PDW and P-LCR exhibited significant negative correlation with platelet count and PCT values, with MPV showing a significant but weak positive correlation with hematocrit.

Conclusion: Monitoring platelet index trends over the disease course, in conjunction with other parameters, can serve as an indirect indicator of the bone marrow response to thrombocytopenia. Falling trends of MPV, PDW, and P-LCR with low platelet counts could suggest a deteriorating patient course and may warrant more vigilant monitoring.

## Introduction

Dengue has emerged as the most widespread and rapidly spreading vector-borne disease in the world [1], with the vast majority of dengue cases being children less than 15 years of age [2]. Many clinical and laboratory parameters have been used to predict the severity of infection, and the management is based on the WHO 2009 revised case classification [3]. This system takes into account certain warning signs and features like severe plasma leakage, bleeding, and organ impairment into consideration [4].

Thrombocytopenia, an important characteristic of dengue viral infection, is observed both in mild and severe dengue; however, the lowest platelet counts are often encountered in severe cases [5]. Suppression of the bone marrow, excessive consumption of platelets, antibody-mediated platelet destruction, complement-mediated lysis, and apoptosis are all responsible for the thrombocytopenia [6]. In addition, platelet indices like mean platelet volume (MPV), platelet distribution width (PDW), and platelet large cell ratio (P-LCR) have also been investigated as prospective platelet activation indicators [7]. In response to the falling platelet counts, immature platelets are thrown into the circulation by the activated bone marrow, resulting in an increased MPV [8]. Conversely, reduced MPV levels are correlated with bone marrow suppression. Platelet activation is also associated with changes in the morphology of platelets, which manifests as increased PDW [9]. Plateletcrit (PCT), an indicator of the total platelet mass, thus denotes consumption of platelets when reduced, and is elevated in the recovery phase, corresponding to a rise in platelet count.

Platelet indices are often reported by most automatic and semi-automatic cell counters used in most hospitals [6]. Despite advances in diagnostic modalities and treatment strategies, there is limited data on dengue fever in the pediatric population and its relation with these platelet parameters. There is a potential for using these indices, in combination with other parameters, in the management of pediatric dengue cases. This study was thus planned to evaluate platelet indices across the range of disease severity, and understand their importance in terms of their trends over the course of the disease among children.

Thus, this prospective observational study was conducted to evaluate hematological parameters, particularly platelet indices, analyze their longitudinal trends, and determine their association with disease severity stratification among children admitted with confirmed dengue fever. The secondary objective was to study the inter-relationship between the various hematological parameters in dengue.

This article was previously presented as a poster at the Indian Public Health Association Conference (IPHACON) on March 23, 2025, in Belagavi, Karnataka, India. It was also presented as an original research article at the Vedam Scientific Conference on September 15, 2024, at Kasturba Medical College, Manipal, Karnataka, India [10].

## Materials and methods

This was a prospective observational study conducted in the Pediatrics department of Belagavi Institute of Medical Sciences (BIMS) Civil Hospital, Belagavi, Karnataka, India, during the peak dengue transmission months of July, August, and September 2024. The study was approved by the BIMS Institutional Ethics Committee (approval number: BIMS-IEC/337/2024-25, dated July 24, 2024). Written informed assent (from children older than 12 years) and consent (from guardians of children younger than 12 years) were obtained.

Study population

Children aged 0-18 years admitted to the pediatric inpatient ward or ICU with clinical features of dengue fever as per WHO 2009 guidelines [3], and diagnosis confirmed by enzyme-linked immunosorbent assay (ELISA) testing for NS1 antigen and/or IgM antibody, were included in the study. Those with a previously known history of thrombocytopenia, with fever and a positive Widal test, and evidence of malarial parasite on peripheral smear were excluded from the study.

The sample size was calculated using the formula: $n = \frac{z^2pq}{d^2}$, ehere p = prevalence of pediatric dengue cases = 17.62% (prevalence of pediatric dengue cases according to a previous study [11]), q = (100 - p) = (100- 17.62) = 82.38%, d = absolute error = 10%, and z = 1.96. 

**Figure 1:**
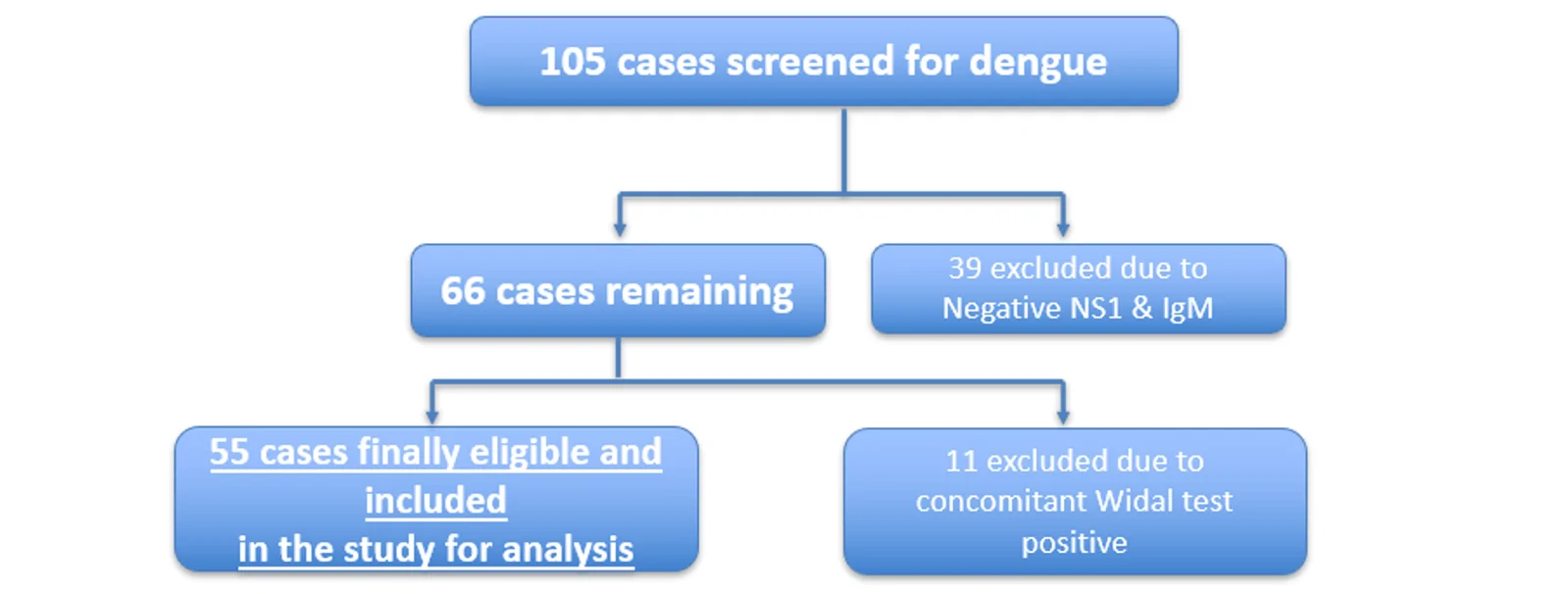
Participant flow diagram

Thus, the final cohort comprised 55 pediatric participants (Figure 1).

Data collection

Clinical and laboratory-based data were collected using predesigned, pretested, structured proformas developed by the authors. Data included demographic details, presenting symptoms, presence of warning signs, and features of severe dengue. Hematological parameters, including platelet indices (platelet count, MPV, PDW, P-LCR, PCT), were recorded using the Erba H360 Hematology Analyzer (Erba Mannheim, Mannheim-Rheinau, Germany) on the first three consecutive days of admission.

Statistical analysis

Statistical analysis was done using IBM SPSS Statistics for Windows, version 23.0 (IBM Corp., Armonk, New York, United States), and data were tested for normality. For normally distributed data, the ANOVA test was used for comparing the indices among the three dengue severity groups, and correlation analysis was done using the Pearson correlation test. Kruskal-Wallis test and Spearman correlation test were used for non-normal data distribution. Paired "t"-test and Wilcoxon Signed-Rank Test were used for analysis of the trends of platelet indices for normally and non-normally distributed data, respectively. The significance level was set at 5%. 

## Results

Out of the 55 study participants, 38 (69%) tested positive for NS1 antigen, while 17 (30.9%) were IgM positive; 15 (27.3%) showed both NS1 and IgM positivity. The cohort exhibited a male predominance (male-to-female ratio of 1.85:1), with adolescents (12-18 years) representing the largest demographic subset (n=31, 56.3%), followed by children aged 6-12 years (n=17, 30.9%) and 0-6 years (n=7, 12.7%). A total of 22 (40%) study participants belonged to rural areas. Fever (2-7 days duration) was universally observed (n=55, 100%), followed by nausea/vomiting (n=46, 83.6%) and headache (n=37, 67.3%). Thrombocytopenia was documented in 38 (69%) of the total participants.

Patients were classified according to the WHO 2009 Revised Case Classification of Dengue [3] based on the worst status during hospitalization. This classification system encompasses warning signs along with features of severe dengue, and 27 (49%) participants had dengue without warning signs (DF-1), 20 (36.3%) had dengue with warning signs (DF-2), and eight (14.5%) had severe dengue (DF-3). The most prevalent warning sign was abdominal pain/tenderness (n=23, 41.8%), followed by a rising hematocrit (n=16, 29%). Severe dengue manifestations included fluid accumulation with respiratory distress (n=5, 62.5%), severe liver involvement (alanine aminotransferase (ALT)/aspartate aminotransferase (AST) >1000 IU/dl; n=4, 50%), and shock (n=3, 37.5%).

The median values of platelet count and PCT in the DF-1 group were 1,56,500 cells/mm^3^ and 0.14%, 85,000 cells/mm^3^ and 0.06% in the DF-2 group, and 66,000 cells/mm^3^ and 0.06% in the DF-3 group, respectively. Median values of platelet count and PCT were lowest in the severe dengue group (DF-3), with concomitantly higher mean values for MPV, PDW, and P-LCR. However, comparison across the three severity groups did not yield a statistically significant difference for any of the tested platelet indices upon admission (Table 1).

**Table 1:** : Comparison of platelet indices among the three groups on the day of admission P-value <0.05 is considered significant; ^*^Kruskal-Wallis test used for platelet count and PCT (non-normal data distribution); ^#^ANOVA used for MPV, PDW, and P-LCR (normal data distribution) Groups were formed as per WHO 2009 Classification of Dengue [3]: DF-1: Dengue without warning signs; DF-2: Dengue with warning signs; DF-3: Severe dengue PCT: plateletcrit, MPV: mean platelet volume, PDW: platelet distribution width, P-LCR: platelet large cell ratio; IQR: interquartile range

Platelet indices		DF-1 (n=27)	DF-2 (n=20)	DF-3 (n=8)	Test statistic	P value
Platelet count (cells/mm^3^)	Mean ± SD	166458. 33 ± 114,151.73	148000 + 125126	109166.67 + 97421.59	1.33*	0.514
	Median (IQR)	156500 (138028, 308528)	85000 (143140, 371140)	66000 (92136, 250886)		
Plateletcrit (%)	Mean ± SD	0.15 ± 0.09	0.12 ± 0.11	0.11 ± 0.1	0.924*	0.63
	Median (IQR)	0.14 (0.08, 0.26)	0.06 (0.02, 0.2)	0.06 (0.02, 0.19)		
MPV (fL)	Mean ± SD	9.27 ± 0.9	9.37 ± 0.91	9.58 ± 0.56	0.326^#^	0.724
PDW (%)	Mean ± SD	15.37 ± 1.95	15.5 ± 1.39	15.77 ± 1.53	0.134^#^	0.875
P-LCR (%)	Mean ± SD	25.19 ± 6.82	24.96 ± 7.32	26.92 ± 3.5	0.201^#^	0.819

**Figure 2:**
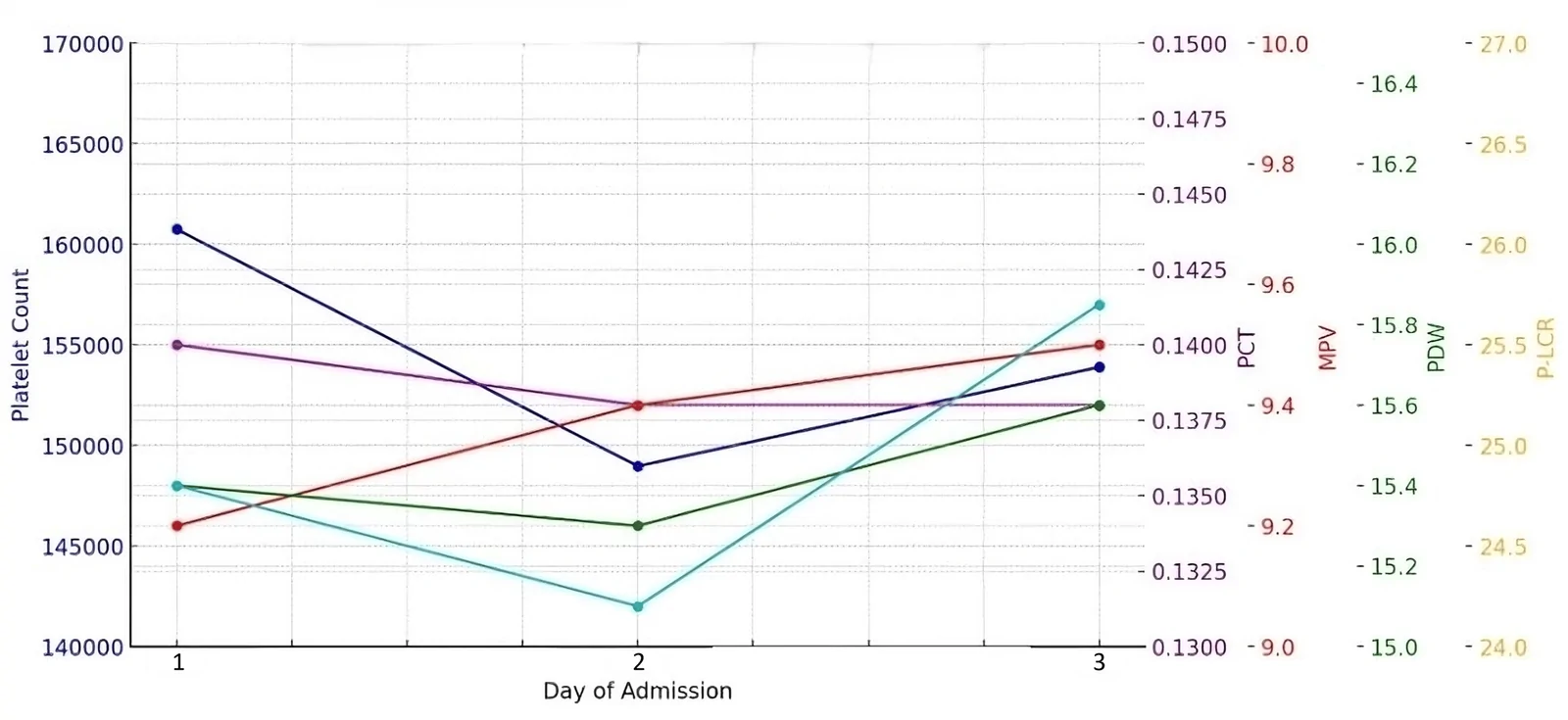
Trends of platelet indices on the first three days of admission in DF-1 group Units: Platelet count: cells/mm^3^, PCT: %, MPV: fL, PDW: %, P-LCR: % MPV and P-LCR follow a similar trend and are represented by a single line graph For analyzing the significance of the trends, a paired t test was used for MPV, PDW, and P-LCR (normal data distribution), and Wilcoxon Signed-Rank Test was used for platelet count and PCT (non-normal data distribution) MPV: mean platelet volume; PDW: platelet distribution width; P-LCR: platelet large cell ratio; PCT: plateletcrit; DF-1: Dengue without warning signs

The platelet indices on the first three days of admission were analyzed (Figure 2). DF-1 group demonstrated a statistically significant rising trend in MPV (p=0.049) and a significant rise in PDW from Day 2 to Day 3 (p=0.044).

**Figure 3:**
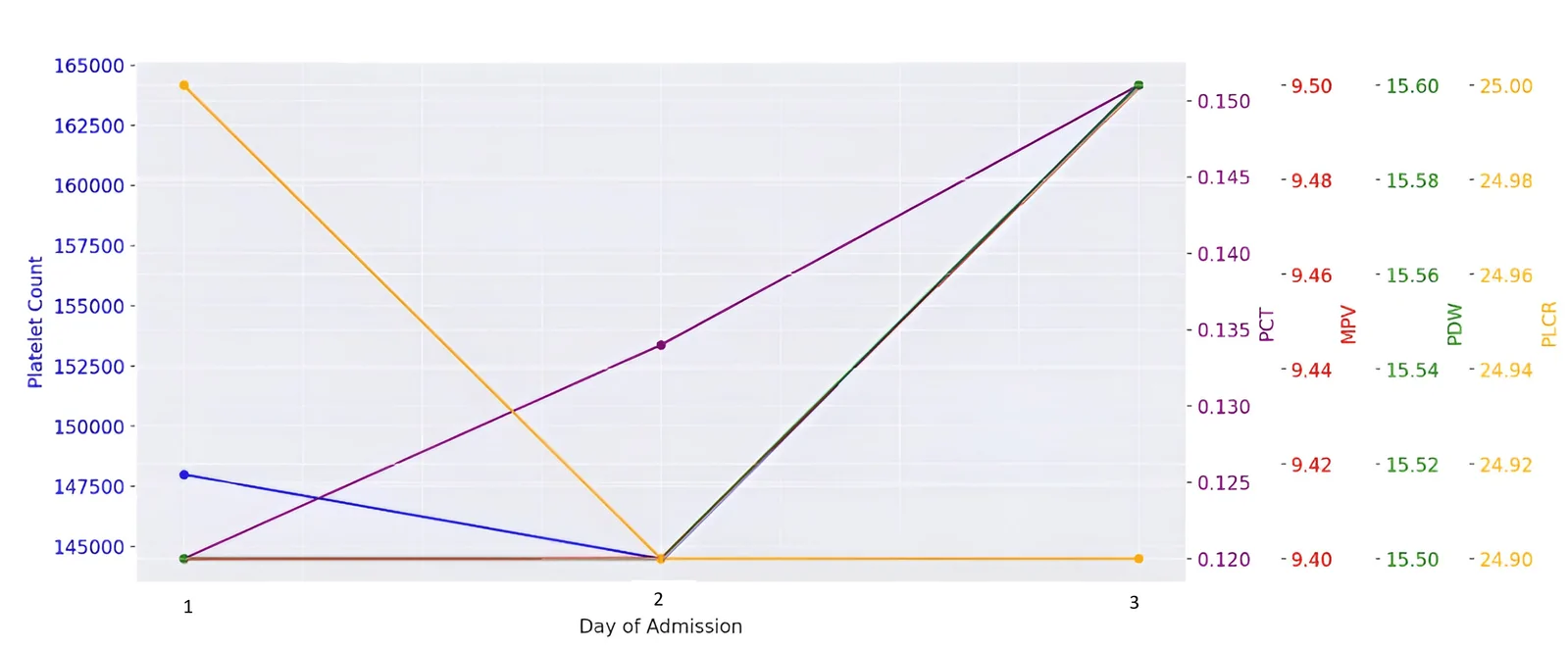
Trends of platelet indices on the first three days of admission in DF-2 group Units: Platelet count: cells/mm^3^, PCT: %, MPV: fL, PDW: %, P-LCR: % MPV and PDW follow a similar trend, and are represented by a single line graph For analyzing the significance of the trends, paired "t" test was used for MPV, PDW & P-LCR (normal data distribution), and the Wilcoxon Signed-Rank Test was used for Platelet count & Plateletcrit (non-normal data distribution) MPV: mean platelet volume; PDW: platelet distribution width; P-LCR: platelet large cell ratio; PCT: plateletcrit; DF-2: Dengue with warning signs

Similarly, the DF-2 group showed upward trending values for platelet count, PCT, MPV, and PDW over the course of the disease. Also, a significant increase in mean platelet count was observed from day 2 to 3 (p=0.029), as shown in Figure 3.

**Figure 4:**
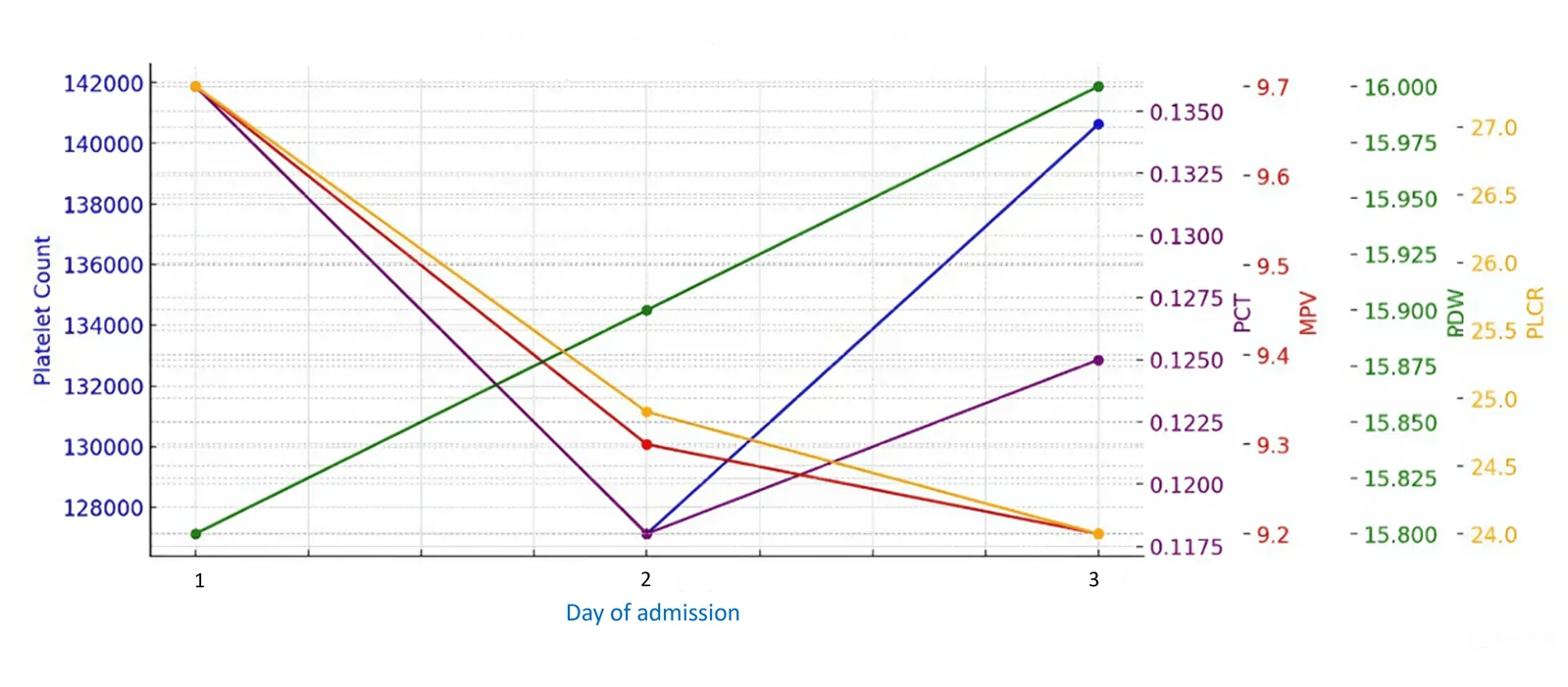
Trends of platelet indices on the first three days of admission in DF-3 group Units: Platelet count: cells/mm^3^, PCT: %, MPV: fL, PDW: %, P-LCR: % For analyzing the significance of the trends, paired "t" test was used for MPV, PDW & P-LCR (normal data distribution), and the Wilcoxon Signed-Rank Test was used for Platelet count & Plateletcrit (non-normal data distribution) MPV: mean platelet volume; PDW: platelet distribution width; P-LCR: platelet large cell ratio; PCT: plateletcrit; DF-2: Severe dengue

Among participants in the DF-3 group, we observed a falling trend in MPV and P-LCR, alongside a progressively rising PDW (Figure 4). These trends were not statistically significant.

Overall, we found no statistically significant difference in the mean and median values of platelet indices among the three dengue severity groups. MPV showed a rising trend in the DF-1 and DF-2 groups, while it trended downwards in the DF-3 group. The trend in the DF-1 group was statistically significant. Although not significant, P-LCR showed a tendency to rise over the course of admission in the DF-1 group; however, a falling trend was observed in the DF-2 and DF-3 groups. 

Correlation analysis revealed several statistically significant relationships between the hematological parameters, as shown in Table 2. A strong positive correlation was observed between platelet count and PCT (r=0.876, p<0.001). PDW, P-LCR, hemoglobin (Hb), and hematocrit (HCT) all exhibited significant negative correlations with platelet count and PCT values. MPV and PDW correlated positively with P-LCR. MPV also showed a weak but significant positive correlation with HCT (r=0.277, p=0.041).

**Table 2:** Correlation between various platelet indices and hematological parameters Data is represented as r (Spearman correlation coefficient, except MPV and Hb) ^#^ Pearson correlation test used for MPV and Hb (normal data distribution); Spearman correlation test used for all the other indices (non-normal data distribution); * statistically significant (p value <0.05) PCT: plateletcrit; MPV: mean platelet volume; PDW: platelet distribution width; P-LCR: platelet large cell ratio; Hb: hemoglobin; HCT: hematocrit

Indices (r, p)	Platelet count (cells/mm^3^)	PCT (%)	MPV (fL)	PDW (%)	PLCR (%)	Hb (g/dL)	HCT (%)
Platelet count (cells/mm^3^)	1	0.876, <0.001*	-0.262, 0.053	-0.492, <0.001*	-0.362, 0.007*	-0.492, 0.001*	-0.456, <0.001*
PCT (%)	-	1	-0.214, 0.116	-0.454, <0.001*	-0.324, 0.016*	-0.388, 0.003*	-0.388, 0.003*
MPV (fL)	-	-	1	0.205, 0.314	0.529, <0.001*	0.213, 0.118 ^#^	0.277, 0.041*
PDW (%)	-	-	-	1	0.366, 0.006*	0.082, 0.551	0.118, 0.39
PLCR (%)	-	-	-	-	1	0.206, 0.131	0.211, 0.122
Hb (g/dL)	-	-	-	-	-	1	0.904, <0.001*

## Discussion

Our study, conducted during the peak monsoon months of July to September in Karnataka, coincided with a notable surge in dengue fever cases. This seasonal trend contrasts with the findings of Makwana et al. [11], who reported peak transmission during the post-monsoon season in Western Rajasthan. In our study, 22 (40%) patients with confirmed dengue fever were from urban areas, whereas Makwana et al. reported a much higher urban representation (80%) [11]. A male predominance was noted in our cohort (male-to-female ratio 1.85:1), in contrast to the more balanced gender distribution (55% male) observed by Makwana et al. [11]. The majority of participants were adolescents aged 12-18 years (n=31, 56%), differing from the observations of Jayashree et al. [6], Shwetha et al. [12], and Sontakke et al. [13], who reported the highest incidence among younger children (6-10 years).

The diagnosis was confirmed serologically in children presenting with classic symptoms. NS1 antigen was positive in 38 (69%) cases, indicating early-phase infection, while 17 (30.9%) were IgM positive. Fever was the most common presenting symptom, followed by nausea/vomiting and headache. Thrombocytopenia was present in 38 (69%) study participants and was most severe among those with severe dengue, aligning with Makwana et al. [11], who reported a mean platelet count as low as 22,360 ± 30.70 cells/mm³ in severe cases.

The most prevalent warning sign was abdominal pain/tenderness (n=23, 41.8%), followed by a rising hematocrit (n=16, 29%). This contrasts with Pothapregada et al., who found persistent vomiting (76.6%) to be the predominant warning sign [14]. Severe dengue developed in eight (15%) cases, with fluid accumulation and respiratory distress (n=5, 62.5%) being the most common severe manifestations. This differs from Pothapregada et al., who reported shock as the most frequent complication (40.5%), compared to three participants (37.5%) developing shock in our cohort among participants with severe dengue [14].

Although several risk prediction models have been proposed for early stratification and management of pediatric dengue [15,16], platelet indices that are readily available in most laboratories have seldom been utilized in clinical decision-making [13]. In our study, we evaluated the role of these indices in relation to disease severity.

On the day of admission, participants in the severe dengue group had lower platelet counts and PCT, accompanied by higher MPV, P-LCR, and PDW values in comparison to the other groups. Although not statistically significant, the release of larger immature platelets into the circulation in response to the thrombocytopenia is represented by a higher value of these indices. These findings are consistent with Nehara et al., who also reported significantly lower platelet counts and plateletcrit, with higher PDW in dengue hemorrhagic fever cases [17].

In the dengue group without warning signs, based on the significantly rising trend of MPV and PDW from day 1 to day 3 of admission, we could hypothesize a possible robust bone marrow response [6] to thrombocytopenia and thus a potential for faster recovery. Similarly, in the dengue group with warning signs, PCT, MPV, and PDW also demonstrated an upward, although not significant, trend over the disease course. Conversely, participants who progressed to severe dengue with manifestations such as shock, respiratory distress, and severe hepatic involvement showed a declining trend of MPV and P-LCR. While it was not statistically significant, this could suggest direct bone marrow suppression by the dengue virus in severe disease [6], and can be an indication for more aggressive monitoring. However, studies with longer follow-up periods are required to create stronger evidence. 

In our correlation analysis, PDW and P-LCR increased significantly with falling platelet counts and PCT, similar to the findings by Jacob et al. [9] and Sontakke et al. [13]. No significant correlation between MPV and platelet count was observed. This aligned with the study by Ezhilarasu et al., where they did not observe a significant change in MPV with rising platelet counts during the critical phase of dengue [18]. Furthermore, we found that a significantly rising HCT correlated with a higher MPV. Although this was a weak positive correlation, it supports the link between hemoconcentration, thrombocytopenia, and third-space fluid loss, as also reported by Nanayakkara et al. [19]. 

While several studies have correlated platelet indices with platelet count, few have examined their association with clinical disease severity. This study provides a framework for further large-scale studies to incorporate these indices into risk stratification guidelines.

The limitations of the study are the small sample size (with only eight participants in the severe dengue group), single-center design, variation in the day of illness on admission, and a short follow-up period. As only more symptomatic or severe cases of pediatric dengue admitted from a single hospital were included, a potential selection bias is likely, as the sample may not be representative of the broader pediatric dengue population. Addressing these limitations in a multicentric study with a bigger sample size would better analyze these trends in relation to dengue severity.

## Conclusions

This study explores the potential of using platelet index trends as adjuncts in the vigilant management of pediatric dengue patients presenting with thrombocytopenia. The dynamic changes in these indices provide insights into the underlying pathophysiological processes. A rising MPV and PDW trend in milder cases could be suggestive of an adequate restorative bone marrow response. Also, the observation of falling MPV and P-LCR trends in severe dengue cases, particularly in the context of persistent thrombocytopenia, may be indicative of direct viral-mediated bone marrow suppression. These specific trends, in addition to validated clinical criteria, should trigger heightened clinical vigilance and may warrant more intensive monitoring.

We recommend further research into the routine monitoring of platelet indices alongside the standard hematological workup in pediatric dengue management, and the potential for utilization of these dynamic markers for early risk stratification. Furthermore, larger multicentric prospective studies are imperative to standardize the use of these indices, validate their predictive power across diverse populations, and explore their potential integration into the standard dengue management guidelines.
